# Biomimetic Enamel Remineralization in Pediatric Dentistry: From Biological Principles to Clinical Evidence

**DOI:** 10.7759/cureus.117599

**Published:** 2026-10-07

**Authors:** Randa M Ibrahim

**Affiliations:** 1 Department of Orthodontics and Pediatric Dentistry, College of Dentistry, Qassim University, Buraydah, SAU

**Keywords:** bioactive glass, biomimetic enamel remineralization, casein phosphopeptide-amorphous calcium phosphate (cpp-acp), cpp-acp, enamel remineralization, nano-hydroxyapatite, pediatric dentistry, self-assembling peptide p11-4

## Abstract

Remineralization of early enamel lesions is a crucial component of contemporary caries management in pediatric dentistry. Biomimetic approaches aim to support enamel repair by reproducing some features of natural mineralization. This narrative review presents the biological basis, mechanisms of action, and current literature on calcium-phosphate systems, nano-hydroxyapatite (nHAp), self-assembling peptide P11-4, and bioactive glasses (BG), with particular emphasis on pediatric dentistry. Although these approaches share the goal of restoring mineral to demineralized enamel, their mechanisms differ and sometimes overlap. Calcium-phosphate systems provide a reservoir of bioavailable calcium and phosphate ions at the tooth surface, mimicking the mineral-supplying function of oral fluids. nHAp resembles enamel apatite crystals in composition and nanoscale dimensions and acts as a mineral template for nucleation and further crystal growth. P11-4 self-assembles within the enamel lesion into a fibrillar scaffold that mimics the mineralization-guiding function of enamel matrix proteins and provides sites for mineral nucleation. Ion exchange, occurring when BG contacts oral fluids, creates a physicochemical environment at the enamel surface that promotes calcium-phosphate precipitation and subsequent apatite formation. Experimental and clinical studies support the remineralizing potential of these approaches. However, pediatric clinical evidence is more limited for some materials than for others, and differences in formulations, study designs, outcome measures, and follow-up periods complicate interpretation of the available literature. Comparisons with conventional fluoride therapy have not consistently demonstrated superior outcomes. Further well-designed pediatric studies are needed to establish clinical effectiveness and determine whether these approaches offer additional benefits in practice. Other emerging biomimetic approaches remain largely experimental. Biomimetic remineralization may therefore offer additional options for the management of early enamel caries in children, while complementing rather than replacing established fluoride-based preventive measures.

## Introduction and background

Advances in preventive dentistry have produced contemporary approaches to enamel remineralization and caries control [1,2]. Nevertheless, dental caries remains a major clinical and epidemiological challenge in pediatric populations; a recent systematic review and meta-analysis estimated the global prevalence of early childhood caries at 49% [3]. Caries management has increasingly shifted towards preventive and minimally invasive strategies that emphasize the preservation of natural tooth structure [2].

Fluoride remains a cornerstone of caries prevention, supported by a substantial body of clinical evidence. However, fluoride-mediated remineralization may be greater at the lesion surface and may not achieve complete mineral recovery within deeper subsurface regions. Moreover, concerns regarding fluorosis risk in young children further highlight the need for complementary preventive strategies [2,4].

In response to these limitations, biomimetic enamel remineralization has emerged as an approach to enamel repair that aims to emulate key physicochemical conditions involved in natural enamel formation and potentially allow deeper and more organized mineral deposition than conventional remineralizing approaches [2,4]. Several mechanisms are involved, including stabilizing calcium and phosphate ions, providing apatite-like crystal templates, recreating extracellular matrix functions that guide crystal nucleation, or establishing a local environment favorable for crystal growth [1,2,4-6]. These strategies are continuously expanding, resulting in a diverse range of biomimetic remineralizing agents with different mechanisms of action and varying levels of clinical evidence [6,7]. The nature and extent of this biomimicry vary among materials, as each reproduces different features of the natural mineralization process [4,8].

Children represent a unique target population for enamel remineralization therapies because of both biological and clinical factors. Primary tooth enamel is generally thinner and less mineralized, with lower calcium and phosphorus levels and greater permeability than permanent enamel [9,10]. These features contribute to increased porosity and lower microhardness in primary enamel, which might lead to more rapid caries development and differences in response to preventive and therapeutic interventions [10-12]. Similarly, newly erupted permanent teeth are vulnerable to demineralization because post-eruptive enamel maturation is incomplete during the early eruption period [10,13]. These anatomical characteristics, together with the high prevalence of early childhood caries and behavioral factors that influence disease progression, may create a relatively limited opportunity for early enamel lesions to be successfully managed before cavitation develops [10,14,15]. Thus, biomimetic remineralization may be useful in pediatric dentistry as a non-invasive strategy for managing early caries lesions.

Several other approaches to biomimetic enamel remineralization are being investigated, such as enamel matrix proteins and derived peptides, polyamidoamine dendrimers, and newer biomimetic scaffolds. These emerging approaches explore additional ways of reproducing or supporting processes involved in enamel mineralization [16-19]. More recently, peptide-based approaches have expanded toward multifunctional designs that combine remineralization with other biological or material functions [20].

This narrative review aims to examine the biological rationale, mechanisms of action, and current experimental and clinical evidence for calcium-phosphate systems, nano-hydroxyapatite (nHAp), self-assembling peptide P11-4, and bioactive glasses (BGs), with emphasis on their potential role in pediatric dentistry and the evidence available for primary and young permanent teeth.

## Review

Literature search

A narrative literature search was conducted using PubMed, Scopus, Web of Science, and Google Scholar, with combinations of terms related to biomimetic enamel remineralization, pediatric dentistry, primary teeth, casein phosphopeptide-amorphous calcium phosphate (CPP-ACP), functionalized tricalcium phosphate (fTCP), nHAp, self-assembling peptide P11-4, and BG. Clinical trials, experimental studies, and review articles were considered, with particular attention to evidence relevant to pediatric dentistry. As this was a narrative review, no formal systematic selection, risk-of-bias assessment, or quantitative synthesis was performed.

Enamel biomineralization

Tooth enamel is a highly mineralized tissue composed of approximately 96% inorganic material, predominantly hydroxyapatite, 1% organic matrix, and 3% water. Its crystallites are hierarchically organized into rod and interrod structures extending from the dentino-enamel junction to the tooth surface [21]. Enamel formation, or amelogenesis, is a highly regulated process that proceeds through secretory, transition, and maturation stages. During these stages, ameloblasts coordinate crystal nucleation, elongation, and maturation through regulated ion transport and controlled secretion and degradation of enamel matrix proteins [21,22]. However, once enamel is mature, ameloblasts are lost, and enamel becomes an acellular tissue incapable of biological regeneration. Consequently, mineral loss caused by dental caries, erosion, or developmental defects cannot be repaired through tissue regeneration but depends on remineralization of the residual enamel scaffold [22].

Dental caries represents a dynamic process characterized by repeated cycles of demineralization and remineralization. Although saliva and fluoride contribute to natural mineral recovery, their ability to restore the original structure of established subsurface lesions is limited. These biological constraints have stimulated the development of biomimetic strategies designed to reproduce selected aspects of natural enamel mineralization and, thereby, enhance enamel repair [2,6,23].

Contemporary biomimetic strategies for enamel remineralization in pediatric dentistry

Biomimetic remineralizing materials differ in their composition and mode of action, although there is some overlap in how they promote enamel repair. The principal approaches currently relevant to pediatric dentistry are discussed below.

Calcium-Phosphate Based Systems

Calcium-phosphate-based systems constitute a principal class of biomimetic enamel remineralization strategies [1,4,8]. Their rationale is based on the dynamic equilibrium between enamel demineralization and remineralization. Under physiological conditions, oral fluids provide calcium and phosphate ions, the principal ionic constituents of hydroxyapatite, thereby maintaining mineral supersaturation and supporting crystal stability and enamel repair. Repeated acidogenic challenges from cariogenic biofilms disrupt this equilibrium and promote the loss of mineral ions from enamel [24,25]. Calcium-phosphate-based technologies function as biomimetic ion delivery systems by increasing the availability of calcium and phosphate ions and thereby emulating the natural ionic conditions that favor enamel remineralization [1,5,8,26]. Among these, two major approaches are CPP-ACP and fTCP [6,27].

Early work by Reynolds on CPP-ACP was an important step in its development as an enamel remineralizing agent [28]. CPP-ACP formulations have been extensively studied, both in laboratory models and in clinical applications, including those involving children [5,29]. This material is a bioactive, casein-derived complex in which casein phosphopeptides stabilize nanoclusters of amorphous calcium phosphate, thereby maintaining elevated concentrations of calcium and phosphate ions in a soluble, bioavailable state. These nanocomplexes localize within the dental plaque, the acquired pellicle, and on the enamel surface, forming a dynamic mineral reservoir capable of releasing calcium and phosphate ions during acidic episodes. This mechanism mimics the mineral-supplying function of oral fluids and facilitates the diffusion of calcium and phosphate into subsurface enamel lesions, thereby promoting the formation of hydroxyapatite crystals within demineralized enamel [6,26]. Fluoride may be incorporated into the stabilized complex, enabling the simultaneous delivery of calcium, phosphate, and fluoride ions. This combination may provide higher remineralization potential than CPP-ACP alone and facilitate fluoride diffusion into deeper regions of subsurface lesions [5,27,30]. fTCP, when combined with fluoride, consists of particles protected by an organic coating that reduces premature interactions between calcium and fluoride within the formulation. Exposure to saliva disrupts this protective barrier, allowing calcium, phosphate, and fluoride to become available at the tooth surface, thereby enhancing acid resistance, fluoride-ion penetration, and enamel remineralization [5,30,31].

CPP-ACP is widely available in various formulations, alone or in combination with fluoride, including toothpastes, mouthwashes, dental creams, chewing gums, and varnishes [6,24,32]. CPP-ACP products generally exhibit good safety and biocompatibility, although rare allergic reactions have been reported [32]. The non-invasive nature and ease of application, through home-use pastes or professionally applied gels, enhance clinical feasibility, particularly in young patients who may have limited tolerance for invasive procedures [32].

Extensive in vitro and in vivo research has demonstrated the remineralizing potential of CPP-ACP [24,33,34]. Notably, several studies have reported that the combined effect of calcium-phosphate-based systems in conjunction with fluoride showed more promising results in enamel remineralization and treatment of early carious lesions [5,35,36]. Additionally, CPP-ACP has been useful in improving the white spot lesions associated with fixed orthodontic treatment [33,37,38]. However, clinical findings are less consistent than laboratory results. Recent systematic reviews, including a 2026 review by Mishra et al. [7], have identified considerable variation in formulations, comparators, outcome measures, and treatment effects, with no consistent superiority of CPP-ACP over conventional fluoride therapy [7,32,39]. Evidence for fTCP is less extensive and has largely involved fluoride-containing formulations, which have shown favorable remineralizing effects, although the independent contribution of fTCP cannot readily be distinguished from that of fluoride [24,30,31].

Several studies have demonstrated the remineralizing effects of CPP-ACP on early carious lesions in primary teeth, both in laboratory models [11,40,41] and in clinical trials [29,42,43]. However, CPP-ACP has not consistently demonstrated superiority over conventional fluoride regimens [26,34,43-45]. In a large randomized clinical trial involving 582 high-caries-risk preschool children followed for 24 months, fluoride varnishes containing CPP-ACP or TCP did not provide a clear additional caries-preventive benefit over conventional 5% sodium fluoride (NaF) varnish [46]. In contrast, a 2025 randomized trial in children aged 10-14 years with white spot lesions demonstrated significant improvement with both CPP-ACP and CPP-ACP with fluoride (CPP-ACPF) using a combined in-office and home-application protocol. The improvements increased over the one-month follow-up, while CPP-ACPF also produced a marked improvement in lesion color; however, the short follow-up and reliance on lesion-level outcomes should be considered when interpreting the findings [47].

*nHAp* *Systems*

Hydroxyapatite has long been studied because of its similarity to the mineral component of bone and teeth. Its use for caries prevention began to emerge in Japan in the 1980s, and advances in nanotechnology later supported the development of nHAp for enamel remineralization [48]. nHAp is regarded as a highly biocompatible, biomimetic, and bioactive material [41,48]. Its chemical composition, crystallinity, and nanoscale dimensions closely resemble those of natural enamel apatite crystallites [6,48]. These dimensions enable effective penetration into enamel microporosities and subsurface lesions formed during early stages of demineralization. nHAp is itself a calcium-phosphate material; however, it is discussed separately from calcium-phosphate systems such as CPP-ACP and fTCP because of its role as an apatite-like mineral template. The nHAp particles serve as nucleation templates that imitate the fundamental mineral building blocks of enamel. They attract calcium and phosphate ions from the surrounding oral environment, promoting their orderly deposition onto the damaged enamel crystal lattice and supporting further crystal growth and integration with the native enamel structure [6,49]. Additionally, the particles aggregate on the enamel surface, forming a layer that functions as a stable mineral reservoir, maintaining a localized supersaturated environment of calcium and phosphate ions [2,48]. The combined subsurface crystal repair and surface protective effects result in increased enamel microhardness and enhanced resistance to repeated acid challenges [11,26]. nHAp is available in several formulations for home use, including toothpastes, gels, and mouthrinses [48], which may facilitate its application in pediatric preventive care.

In vitro studies have provided substantial evidence that application of nHAp-containing formulations can prevent and reverse early enamel caries. These studies have demonstrated that nHAp particles penetrate the porous surface layer of incipient lesions and promote crystal growth, leading to increased mineral recovery, improved microhardness, reduction in lesion depth, and enhanced resistance to subsequent acid challenge [50-52]. In primary enamel, several studies evaluating nHAp dentifrices reported increased surface microhardness in artificial caries lesions created in extracted primary teeth [40,53-55]. A 2026 systematic review of studies on primary teeth concluded that nHAp and fluoridated nHAp (nHAp-F) are effective remineralizing agents, although all 15 included studies were conducted in vitro [41].

Clinical evidence is more limited but has increased in recent years. A systematic review (2025), which included only four clinical trials, one of them in primary teeth, reported greater reductions in white spot lesion size and improvements in mineral density with nHAp toothpaste compared with fluoride toothpaste. However, the interpretation of these findings is limited by factors such as variations in study designs and methods used to evaluate caries progression [56].

In the 2025 randomized clinical trial by El Mansy et al., 10% nHAp significantly improved white spot lesions in children aged 10-14 years over the one-month follow-up [47]. Although these findings support the remineralizing potential of nHAp in early enamel lesions, the short follow-up limits conclusions regarding the persistence of the observed improvement. Evidence over a longer period was reported by Cocco et al. (2025). They conducted a large randomized clinical trial that included 518 children aged four to seven years over a 24-month follow-up period. Toothpastes containing nHAp-F were more effective than standard sodium monofluorophosphate toothpastes in arresting active caries lesions and promoting enamel remineralization [57].

Despite these findings, pediatric clinical evidence remains limited, particularly regarding the long-term effectiveness of nHAp across different age groups, formulations, and conditions of use [56,57].

Self-Assembling Peptide P11-4

Self-assembling peptide P11-4 is one of the most explicit biomimetic approaches to the remineralization of early enamel lesions. Kirkham et al. provided early evidence for the application of P11-4 to enamel repair, showing increased mineral gain in caries-like enamel lesions [58]. This synthetic 11-amino-acid oligopeptide is delivered as a low-viscosity solution, enabling diffusion into the porous subsurface of non-cavitated lesions. Within the lesion microenvironment, P11-4 undergoes hierarchical self-assembly to form a three-dimensional fibrillar scaffold that mimics the mineralization-guiding function of enamel matrix proteins. This scaffold has an affinity for calcium ions, thus facilitating the deposition of calcium and phosphate from the surrounding oral environment and providing nucleation sites for hydroxyapatite formation. By promoting mineral deposition within the lesion body rather than only at the enamel surface, P11-4 provides a matrix-guided biomimetic approach to enamel remineralization [5,8,58-60]. However, although this process is sometimes described as enamel regeneration, P11-4 does not reproduce natural amelogenesis or restore the original biological architecture of enamel. The terms "enamel repair" or "biomimetic remineralization", therefore, provide a more cautious description of its effect [5,59,60].

The remineralization potential of P11-4 has been investigated in both laboratory and clinical studies. A systematic review including 12 in vitro studies reported significant remineralization of artificially demineralized enamel surfaces after the application of P11-4 [61]. Similar positive results were obtained from in vitro studies involving primary teeth [62,63]. P11-4 and fluoride may have complementary effects, as P11-4 provides an intralesional matrix for mineral nucleation, while fluoride promotes mineral precipitation and increases the acid resistance of the newly formed mineral. Accordingly, combining P11-4 with fluoride has been associated with significantly more mineralization than P11-4 alone in vitro [62].

Available evidence indicates generally good tolerability, with no serious treatment-related adverse effects [64,65]. Systematic reviews have evaluated a substantially overlapping group of clinical trials but have reached different levels of confidence in the effectiveness of P11-4. A 2023 systematic review and meta-analysis of eight randomized clinical trials found considerable variation in the results across different methods used to assess remineralization, including laser fluorescence, International Caries Detection and Assessment System (ICDAS) and Nyvad criteria. Due to the heterogeneity of the studies and the high risk of bias in four of the eight trials, the authors considered the evidence insufficient to draw firm conclusions regarding the remineralizing effect of P11-4 with or without fluoride [65]. Another 2023 meta-analysis of six clinical trials reported increased caries arrest and reduced lesion size following P11-4 treatment, although all included trials had an elevated risk of bias [66]. A more recent systematic review, including seven clinical trials, reported P11-4 as a promising non-invasive approach for the management of early caries lesions, with greater subsurface remineralization than fluoride-based regimens, but emphasized heterogeneity and the predominantly short-term nature of the available evidence [67].

Clinical trials involving children are relatively few. A randomized clinical trial by Doberdoli et al. (2020) showed better results for P11-4 combined with fluoride varnish in the treatment of non-cavitated occlusal caries lesions in children and adolescents compared with fluoride varnish alone over a 12-month period [64]. Another clinical trial, involving high caries risk preschool children, reported that intensive application of P11-4 plus fluoride produced favorable esthetic and remineralization effects on white spot lesions in primary teeth compared with fluoride varnishes [29]. More recently, Elaziz et al. (2025) compared P11-4 with arginine-enriched NaF and fTCP-fluoride varnishes for white spot lesions in children aged eight to 12 years. All three interventions showed remineralization reflected by improved lesion color during a follow-up period of nine months, although P11-4 showed less improvement than the other two regimens [68].

Bioactive Glass

BG is a promising material for biomimetic enamel remineralization and caries management. It promotes enamel repair mainly through controlled ion release and in situ apatite formation, recreating a physicochemical environment favorable for mineralization [4,26,69]. The discovery of BG by Hench in 1969 demonstrated that certain glass compositions could form a stable chemical bond with surrounding host tissues [70].

BGs are silica-based biomaterials, most commonly calcium-sodium-phosphosilicate formulations. When the BG is in contact with saliva, sodium ions in the glass are exchanged with hydrogen ions from saliva, producing a localized alkaline environment that counteracts acid-mediated enamel dissolution. Simultaneously, calcium, phosphate, and silicate ions are released into the surrounding medium, creating supersaturation with respect to apatite minerals. This ionic environment promotes deposition of an amorphous calcium phosphate precursor that gradually matures into hydroxycarbonate apatite crystals on the enamel, resembling the mineral composition of natural tooth enamel [6,54,70]. Fluoride‑containing BG formulations encourage the precipitation of fluorapatite crystals, thus enhancing the acid resistance of the remineralized enamel [71]. Nano-BG (nBG) has been developed with a smaller particle size to enhance the remineralization efficiency of conventional BG. The nano-sized particles increase surface reactivity and accelerate ion release, while their small size allows penetration into deeper subsurface enamel lesions [69]. For enamel remineralization, BG has been incorporated into oral-care formulations such as toothpastes and professionally applied varnishes [69].

Experimental evidence supports the remineralizing potential of BG-based materials [69,72]. In primary enamel, conventional BG showed increased mineral density and microhardness recovery in artificial caries-like lesions [73]. Subsequent studies evaluated fluoride-containing formulations and nBG in primary enamel models. Fluoride-incorporated BG toothpastes showed remineralization effects on artificial caries in primary enamel at both surface and subsurface levels [74,75]. In a study by ElZohairy et al. (2026), nBG re-established mineral content under dynamic pH conditions within early enamel lesions induced in primary teeth. Enamel repair involved the deposition of a dense hydroxycarbonate apatite layer that restored surface continuity, while ions released from the nanoparticles diffused into the demineralized subsurface, resulting in a measurable reduction in lesion depth [54].

Clinical studies have also shown remineralizing effects of BG-based formulations in early enamel caries and white spot lesions. However, findings vary according to the formulation and application protocol, and superiority over fluoride has not been consistently demonstrated [7]. A recent systematic review of randomized controlled trials included three BG trials and reported generally favorable remineralization outcomes, although the small number of studies, variation among interventions, and relatively short follow-up periods limit the strength of the available evidence [7]. Among the included trials, Guo et al. reported significant improvement in early enamel lesions following BG treatment compared with placebo, with outcomes comparable to fluoride over the three-month follow-up [76].

Pediatric clinical evidence remains particularly limited. A multicenter randomized controlled trial by Li et al. (2026), including 1063 preschool children, found that a fluoride-free toothpaste containing BG had caries-preventive effects comparable to those of a conventional fluoride toothpaste over 27 months, although both groups showed considerable caries progression [77]. Further clinical studies are needed to determine the effectiveness of BG for enamel remineralization and caries prevention in children.

Comparative evidence in pediatric dentistry

Although the materials discussed in this review are grouped as biomimetic approaches, they reproduce different aspects of natural mineralization [4,8]. Whether closer imitation of biological mineralization translates into greater clinical effectiveness has not yet been established [8]. Comparisons between the approaches are also complicated by differences among the studies. The available evidence therefore needs to be interpreted in the context in which each material has been investigated rather than as a basis for ranking the different approaches.

In artificially demineralized primary enamel, Gayan et al. [11] found significant improvement in surface microhardness with CPP-ACP, nHAp, and NaF. The largest changes were observed with CPP-ACP and NaF, while nHAp showed moderate efficacy, although differences among the three were not statistically significant. Mareddy et al. [78] also studied primary enamel but used an erosion model and reported the greatest surface microhardness recovery with nHAp compared with BG and CPP-ACPF. A different pattern was observed by ElZohairy et al. [54] in artificial carious lesions in primary teeth, where nBG produced the highest calcium content and calcium-to-phosphate ratio and the lowest lesion depth compared with nHAp and NaF plus ACP varnishes, although nBG and nHAp did not differ significantly for some outcomes. A study comparing P11-4 with BG and fTCP also showed that the application protocol can affect the results [79]. These differences may partly reflect variations in the type of mineral loss, formulation and concentration, mode of application, and outcome assessed.

Clinical studies have also produced varied results. In children with white spot lesions, El Mansy et al. [47] reported greater short-term remineralization with nHAp than with CPP-ACP, CPP-ACPF, or fluoride. Other clinical studies have compared individual biomimetic approaches with fluoride. Treatment protocols involving P11-4 produced greater improvement in early occlusal lesions than fluoride varnish alone in children and adolescents [64], while a fluoride-free 7.5% BG toothpaste showed no significant difference in caries incidence or increment compared with an 800-ppm fluoride toothpaste after a 27-month study in preschool children [77].

These findings also highlight the importance of the outcomes assessed across studies. Laboratory measures such as microhardness, mineral content, and lesion depth provide useful information on remineralization, but favorable experimental findings do not necessarily translate into similar clinical effects. This is particularly important when pediatric clinical evidence is limited. In pediatric dentistry, practical factors also need to be considered. Some approaches are intended for repeated home use, while others require professional application or several treatment sessions. Adherence, parental supervision, and child cooperation may therefore affect how easily these approaches can be used in practice. Caries risk, lesion characteristics, and the requirements of the treatment protocol should also be considered when evaluating their use in children.

Emerging approaches

Several emerging biomimetic approaches are currently under investigation. Among these, enamel matrix proteins and derived peptides are of particular interest because of their role in natural enamel formation. Zhong et al. investigated amelogenin, amelotin, and an ameloblastin C-terminal peptide, individually and in combination. The combination produced more organized and aligned hydroxyapatite crystal bundles than the individual components, suggesting that interactions between different enamel matrix components may contribute to mineral organization [16]. More recently, Ding et al. developed CaAm-P19, an amelogenin-derived peptide with hydroxyapatite-binding properties. In demineralized bovine enamel, the peptide bound to the enamel surface, penetrated deeply into the subsurface lesion, and promoted the deposition of ordered hydroxyapatite-like crystals. It achieved microhardness comparable to fluoride and significantly greater mineral-density recovery [17]. Polyamidoamine dendrimers are another scaffold-based approach. Their branched structure can provide sites for mineral nucleation and guide subsequent crystal growth, and experimental studies have shown remineralization of demineralized enamel [18]. Other new systems have also combined organic scaffolds with mineral precursors. Chen et al., for example, investigated a nanocomposite containing silk fibroin, carboxymethyl chitosan, and amorphous calcium phosphate designed to mimic aspects of amelogenesis. This system promoted enamel remineralization and the formation of enamel-like mineral structures in experimental models [80]. Phosphorylated nano chitosan (Pchi-ACP), a newer biomimetic scaffold, has also been evaluated clinically. In a 12-month randomized trial, Pchi-ACP showed greater and more sustained remineralization of white spot lesions than P11-4 and CPP-ACP [19]. A recent study by Shao et al. investigated the use of chondroitin sulfate in a biomimetic enamel remineralization strategy. Chondroitin sulfate influenced mineral precursor assembly and hydroxyapatite crystallization, resulting in a mineralized layer with an organized structure and mechanical properties approaching those of natural enamel [81]. Despite these developments, evidence for the newer biomimetic approaches remains predominantly experimental, with little clinical investigation in primary or young permanent teeth. Their clinical role in pediatric dentistry therefore remains to be established.

Biomimetic approaches may also have applications beyond early caries lesions. Molar-incisor hypomineralization (MIH) is of particular interest. Calcium-phosphate systems, hydroxyapatite, and self-assembling peptides have been investigated in MIH-affected teeth. Their effects on remineralization and hypersensitivity have varied across studies, but questions remain about the durability of these effects [82].

Beyond enamel, biomimetic strategies are also being investigated for dentin remineralization and regeneration of the dentin-pulp complex [83]. Although outside the scope of this review, these applications represent another developing area of biomimetic research in pediatric dentistry.

## Conclusions

Biomimetic approaches provide additional options for managing early enamel caries in children. Calcium-phosphate systems, nHAp, self-assembling peptide P11-4, and BG promote enamel remineralization through different, although sometimes overlapping, mechanisms. Their remineralizing effects have been demonstrated in experimental and clinical studies, but the clinical evidence is not equally developed among the different materials, particularly in children. At present, these approaches are better considered as complementary to fluoride rather than as replacements for established fluoride-based preventive measures.

Further research is needed to determine whether the remineralizing effects observed experimentally are sustained clinically in primary and young permanent teeth. Longer-term pediatric clinical trials should evaluate appropriate formulations and concentrations, application protocols, and treatment frequency, using consistent and clinically relevant outcome measures. Direct comparisons with established preventive therapies will also help determine whether these approaches offer additional clinical benefits.
